# Longitudinal Changes in Retinal Nerve Fiber Layer Thickness Evaluated Using Avanti Rtvue-XR Optical Coherence Tomography after 23G Vitrectomy for Epiretinal Membrane in Patients with Open-Angle Glaucoma

**DOI:** 10.1155/2017/4673714

**Published:** 2017-07-27

**Authors:** Anita Lyssek-Boroń, Adam Wylęgała, Katarzyna Polanowska, Katarzyna Krysik, Dariusz Dobrowolski

**Affiliations:** ^1^Department of Ophthalmology with Pediatric Unit, Santa Barbara Hospital - Trauma Center, Plac Medyków 1, 42-200 Sosnowiec, Poland; ^2^Ophthalmology Clinic Department of Ophthalmology District Railway Hospital, Medical University of Silesia, Panewnicka 65, 40-760 Katowice, Poland

## Abstract

**Objectives:**

The aim of this study is to evaluate longitudinal RNFL thickness changes in patients with open-angle glaucoma (OAG) who underwent pars plana vitrectomy (PPV) with epiretinal membrane (ERM) and ILM removal using OCT RTVue XR 100 Avanti.

**Methods:**

Retrospective analysis of OCT scans of 40 patients who underwent PPV or combined phacovitrectomy with ILM peeling for the idiopathic ERM has been carried out. The patients were divided into two groups for the study: patients with the ERM and OAG and those with ERM without glaucoma. A trend analysis of the RNFL thickness changes in 1 month and 3, 6, and 12 months was created.

**Results:**

At 1 month after surgery, the RNFL thickness increased significantly in the temporalis quadrant from 89.9 *μ*m to 105.7 *μ*m in patients with OAG. Comparison between group with OAG and group without glaucoma showed that the RNFLT in the temporalis quadrant decreased significantly 6 months after the surgery.

**Conclusion:**

Postoperative changes in RNFL thickness appeared to be transient, and there was temporal retardation of the retinal nerve fibers without affecting visual acuity in both groups.

## 1. Background

In the 1970s, Machemer et al. reported the first pars plana vitrectomy (PPV) that revolutionized retinal surgery [1]. Since then, vitreus surgery has evolved considerably because of the related technological innovations. Currently, vitrectomy is the third most frequently performed ophthalmic procedure.

One of the indications for PPV is the idiopathic epiretinal membrane (ERM). This semitransparent membrane located between the internal limiting membrane (ILM) and the vitreus causes macular dysfunction, such as macular distortion or edema, leading to a decrease in visual acuity and metamorphopsia. Retinal surgery releases the tractional forces on the retinal surface. Vitrectomy for ERM removal with or without ILM removal is the standard treatment for such dysfunction [2, 3]. Although PPV for ERM is generally considered a safe procedure, various complications have been reported, including cataract formation, increase in intraocular pressure (IOP) [4, 5], visual field defects [6, 7], and the development of open-angle glaucoma (OAG) [8]. Further, ILM peeling is known to cause a mechanical damage of the retinal nerve fiber layer (RNFL) [4–6].

To the best of our knowledge, no well-designed controlled studies evaluating the long-term changes in RNFL thickness after vitrectomy in patients with OAG have been reported thus far. Further, recently developed spectral domain optical coherence tomography (SD-OCT) allows greater noninvasive detection of RNFL alterations, using low-coherence interferometry [7]. High resolution, rapid imaging, and excellent repeatability of the RNFL thickness measurement make OCT useful in many studies on ocular diseases such as glaucoma and optic neuropathy [8].

In the present study, we used OCT RTVue XR 100 Avanti (Optovue, Fremont, CA, USA) to evaluate longitudinal RNFL thickness changes in patients with OAG who underwent pars plana vitrectomy with ERM and ILM removal.

## 2. Methods

### 2.1. Subjects

This retrospective study included 40 patients (40 eyes) who underwent pars plana vitrectomy or combined phacovitrectomy with ILM peeling for the idiopathic epiretinal membrane between 2015 and 2016 at St. Barbara Regional Specialist Hospital, Sosnowiec, Poland. Patient consent to review their medical records was not required by the Bioethical Committee because of the retrospective nature of this study and because the patient information was sufficiently anonymized. The selected patients were divided into two groups for the analysis: patients with the ERM and OAG diagnosis (group A) and those with ERM without glaucoma (group B). Each patient underwent a complete ophthalmologic examination, including the best-corrected visual acuity testing using Snellen charts, slit-lamp examination, IOP measurement with Goldmann applanation, and SD-OCT, 1, 3, 6, and 12 months postoperatively. The exclusion criteria for all the participants were as follows: preoperative IOP of >21 mm Hg and cup/disc ratio of >0.5, other retinal diseases, history of uveitis or trauma, optic disc abnormality, optic nerve disorder, history of any neuro-ophthalmologic disease, previous intraocular surgery, and myopia > 6 diopters. Patients with OAG (group A) with less than a 24-month history of glaucoma, visual field defects more than MD-2.0 dB, and/or any incidence of increase in IOP of >30 mm Hg and those taking more than one topical antiglaucoma medication were excluded. The best-corrected visual acuity (BCVA) of all eyes was better than 20/40 (logMar 0.30). The characteristics of the vitrectomized eyes included are summarized in Table 1.

### 2.2. Surgical Procedure

All patients signed the informed consent form before undergoing any surgical procedure. 23-gauge, 3-port pars plana vitrectomy was performed on all the eyes by the same vitreoretinal surgeon (A.B.) with the same technique and the same vitreoretinal machine (Constellation, Alcon, Forth Worth, TX, USA). All phakic patients underwent PPV with phacoemulsification and intraocular lens implant. First, a posterior vitreous detachment was created. Next, 0.1 mL of a Brilliant Blue solution was injected over the retinal surface for 1 min. The dyed ERM was removed with forceps, and the staining procedure was repeated to visualize the ILM. ILM was removed with forceps and peeled (2-disc diameter around the macula) in a circular manner. Thereafter, a fluid-air exchange was performed. A corticosteroid and an antibiotic were applied after surgery for 4 weeks. All patients were examined postoperatively at 1, 3, 6, and 12 months.

### 2.3. Optical Coherence Tomography Measurement

**Figure 1:**
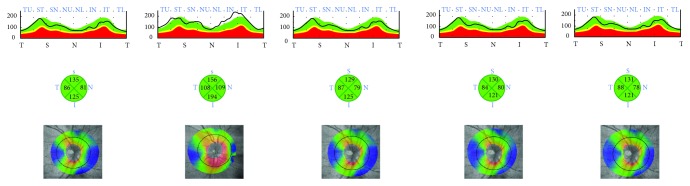
Patient without glaucoma. Color-coded maps of the retinal nerve fiber layer (RNFL) thickness in the four quadrants before and at 1, 3, 6, and 12 months after the surgery (pictures from the left to the right). One month postoperation, the RNFL thickness increased at the inferior nasal and temporal quadrants because of nerve fiber edema. The increase in the RNFL thickness discontinued in the third month postintervention and decreased at 6 months and 12 months after the surgery.

OCT was performed using an RTVue 100 XR Fourier-domain OCT system (Optovue Inc., Fremont, CA, USA). The RTVue system utilized 840 nm near-infrared light, with a 50 nm bandwidth. For the RNFL thickness measurements, a three-dimensional optic disc scan for the definition of the disc margin based on a computer-assisted determination of the retinal pigment epithelium and an optic nerve head (ONH) scan to measure the RNFL thickness within a circle of 4 mm diameter, centered on the preprogrammed disc, were conducted (Figure 1). Each ONH scan contained 12 radial lines and 6 concentric rings, which were used for creating an RNFL thickness map. The measuring circle (920 points) of 3.5 mm diameter was derived from this map after adjusting the sample circle to be centered on the optic disc. ONH was defined at the first visit. Circular OCT tomograms were acquired around the optic disc at a diameter of 3.4 mm. In the statistical analysis, we used four quadrants (superior, inferior, nasal, and temporalis) of the RNFL thickness measurements.

A trend analysis of the RNFL thickness changes is possible and necessitates a minimum of three visits. It is a regression analysis with no assumption of the known test-retest variability. The results are given and graphically presented as the rate of change, 95% confidence interval, and the level of statistical significance (*P* value) [9].

### 2.4. Statistical Analysis

Analyses were performed using statistical package R 3.3.2 (R Project). Descriptive analyses of continuous variables were described with the number of nonmissing observations (N), arithmetic mean, standard deviation (SD), the first quartile, median, the third quartile, minimum, and maximum. To compare the results of continuous variables between groups, a *t*‐test or Mann-Whitney-Wilcoxon test was used. The normality distribution was evaluated using a Shapiro-Wilk test. The significance level was set at 0.05.

## 3. Results

We analyzed 40 eyes of 40 patients, including 21 females and 19 males, who completed a 12-month follow-up. The mean patient age was 70.2 ± 7.1 years (range: 52–81 years). Twenty patients (50%) underwent combined phacovitrectomy, and the other 20 (50%) underwent only vitrectomy. Ten patients had a history of type-2 diabetes mellitus (25%) without diabetic retinopathy and glaucoma, and 12 had a history of hypertension (30%) (patients with glaucoma). Changes in the RNFL thickness after surgery are shown in Table 2.

At 1 month after surgery, the RNFLT increased at the baseline significantly (*P* = 0.009) in the temporalis quadrant. Comparisons between group A with OAG and group B without glaucoma showed that the RNFLT in the temporalis quadrant decreased significantly (*P* = 0.008) only 6 months after the surgery (Table 2). A similar thinning was observed in the nasal quadrant only 3 months after PPV. No significant differences were observed between group A and group B in terms of the total RNFL and the mean RNFLT in the superior and the inferior quadrants.

## 4. Discussion

**Figure 2:**
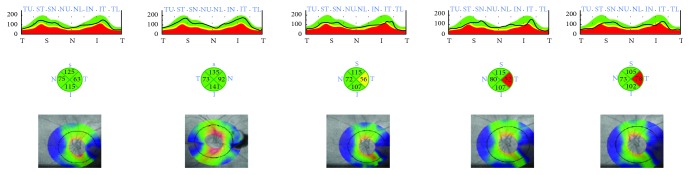
Patient with glaucoma. Color-coded maps of the RNFL thickness in the four quadrants before and at 1, 3, 6, and 12 months after the surgery (from the left to the right). In this patient, postoperative edema of the RNFL is followed by considerably more thinning than that shown in Figure 1.

Over the last decade, OCT has played an important role in ophthalmology for not only detecting but also quantifying the progression of glaucoma. In clinical practice, an assessment of the stability or progression of glaucoma is based on a series of functional tests, including perimetry [10], thickness measurement of RNFL [11, 12]. Measurements made by various Fourier-domain OCTs are of great importance in imaging structural changes observed particularly in the early stages of glaucoma. Early detection of the rapid progression of glaucoma is a decrease in RNFL to 1.5 *μ*m/annum [9]. There are few publications on the analysis of the changes in RNFL after PPV with ERM and ILM in patients with OAG. Reddy et al. described the decrease in RNFL thickness only in the lower quadrants [13], while Kim et al. described it in the upper and lower quadrants [14]. Balducci et al. reported the changes in the upper, lower, and temporal quadrants [15]. After analyzing our results, we have found that there is a statistically significant increase in the RNFL thickness in the first month after temporal quadrant surgery (Figure 2). It is probably associated with edema of the nerve fibers because of the axonal transport disorder leading to apoptosis and atrophy of the ganglion cells [7, 16, 17], which leads to the subsequent RNFL thinning 6 months after the procedure. Another explanation of this mechanism may be dehydration caused by the fluid to air exchange during PPV, toxic retinal damage with gas endotamponade, and mechanical damage during the removal of the vitreous body [7, 16, 17]. Like Gharbiya et al. [16], we did not notice any differences in the RNFL thickness between patients who underwent phacovitrectomy and those who underwent vitrectomy alone. Although our analysis was related to glaucoma patients and controls, we did not observe statistically significant changes in the total RNFL thickness in the upper and lower quadrants in both the groups. Postoperative lesions in RNFL appeared to be transient, and there was temporal retardation of the retinal nerve fibers without affecting visual acuity in both the groups. Since the observation period was 1 year, we concluded that PPV with the removal of the ERM and imaging of the ILM was a safe procedure for glaucoma patients.

## Figures and Tables

**Table 1:** Characteristics of the vitrectomized eyes.

	All patients (*N* = 40)	Group A (*N* = 21)	Group B (*N* = 19)
Age (mean ± SD, years)	70.2 ± 7.1	69.6 ± 7.6	70.9 ± 6.7
Gender			
Male Female	19 21	12 9	7 12
Lens status			
Phakic before PPV Pseudophakic before PPV	20 20	11 10	9 10
Mean BCVA Snellen charts (logMar)		20/40 (0.30)	20/30 (0.20)
IOP before PPV (mean ± SD)		16.25 ± 2.83	14.37 ± 2.46
IOP after PPV (mean ± SD)		17.2 ± 3.22	15.1 ± 4.15

Group A = with glaucoma, Group B = without glaucoma, BCVA: best-corrected visual acuity; IOP: intraocular pressure.

**Table 2:** RNFL thickness changes in group A and group B eyes. Group A: with glaucoma; group B: without glaucoma.

		Baseline	1 month	3 months	6 months	12 months
Total RNFL	Group A					
	RNFLT	93.0 ± 9.08	106.9 ± 9.29	95.8 ± 8.35	91.1 ± 8.42	88.7 ± 9.14
	RNFL changes		13.9 ± 8.38	2.8 ± 7.24	−1.9 ± 4.89	−4.3 ± 4.74
*P* value			*P* = 0.3	*P* = 0.2	*P* = 0.9	*P* = 0.8
	Group B					
	RNFLT	95.8 ± 5.28	107.0 ± 5.36	100.2 ± 4.48	94.6 ± 4.57	91.8 ± 5.24
	RNFL changes		11.2 ± 4.39	4.4 ± 3.72	−1.2 ± 3.55	−3.9 ± 5.80
Superior RNFL	Group A					
	RNFLT	95.5 ± 9.34	111.2 ± 12.56	125.2 ± 12.52	95.8 ± 9.86	94.6 ± 11.83
	RNFL changes		15.7 ± 8.04	7.3 ± 8.45	0.2 ± 4.96	−1.0 ± 5.60
*P* value			*P* = 0.96	*P* = 0.58	*P* = 0.9	*P* = 0.93
	Group B					
	RNFLT	109.4 ± 9.23	125.2 ± 9.83	115.4 ± 6.86	109.5 ± 6.56	106.8 ± 7.01
	RNFL changes		15.8 ± 10.10	6.0 ± 6.45	0.2 ± 6.16	−2.5 ± 7.35
Inferior RNFL	Group A					
	RNFLT	101.3 ± 14.21	117.1 ± 14.57	107.4 ± 13.16	98.7 ± 15.14	96.6 ± 14.84
	RNFL changes		15.8 ± 10.78	6.1 ± 12.10	−2.6 ± 8.11	−4.7 ± 8.26
*P* value			*P* = 0.35	*P* = 0.9	*P* = 0.36	*P* = 0.46
	Group B					
	RNFLT	111.1 ± 9.15	124.1 ± 8.00	117.1 ± 7.58	110.3 ± 8.03	108.9 ± 9.03
	RNFL changes		12.9 ± 8.02	6.0 ± 6.77	−0.8 ± 3.27	−2.2 ± 4.59
Nasalis RNFL	Group A					
	RNFLT	81.5 ± 7.81	91.9 ± 7.42	83.4 ± 7.44	82.9 ± 7.681.4 ± 4.86	82.7 ± 8.37
	RNFL changes		10.4 ± 5.96	1.9 ± 5.66		1.2 ± 6.45
*P* value			*P* = 0.1	**P** = 0.03	*P* = 0.74	*P* = 0.49
	Group B					
	RNFLT	77.1 ± 7.94	85.7 ± 6.08	82.7 ± 5.55	79.8 ± 6.75	79.7 ± 5.58
	RNFL changes		8.6 ± 4.36	5.6 ± 4.37	2.7 ± 4.93	2.6 ± 6.24
Temporalis RNFL	Group A					
	RNFLT	89.9 ± 16.49	105.7 ± 18.11	90.7 ± 14.19	85.8 ± 15.10	81.1 ± 15.29
	RNFL changes		15.8 ± 6.7	0.8 ± 9.90	−4.1 ± 7.02	−8.8 ± 7.12
*P* value			**P** = 0.009	*P* = 0.95	**P** = 0.008	*P* = 0.14
	Group B					
	RNFLT	85.8 ± 9.9	92.5 ± 11.22	85.7 ± 8.15	78.9 ± 7.65	71.9 ± 10.09
	RNFL changes		6.7 ± 4.78	−0.1 ± 6.37	−6.8 ± 5.15	−13.9 ± 10.90

## References

[1] Machemer R., Buettner H., Norton E. W., Parel J. M. (1971). Vitrectomy: a pars plana approach. *Transactions - American Academy of Ophthalmology and Otolaryngology*.

[2] Schweitzer C., Delyfer M. N., Colin J., Korobelnik J. F. (2009). 23-gauge transconjunctival sutureless pars plana vitrectomy: results of a prospective study. *Eye*.

[3] Wong J. G., Sachdev N., Beaumont P. E., Chang A. A. (2005). Visual outcomes following vitrectomy and peeling of epiretinal membrane. *Clinical and Experimental Ophthalmology*.

[4] Weinberg R. S., Peyman G. A., Huamonte F. U. (1976). Elevation of intraocular pressure after pars plana vitrectomy. *Albrecht von Graefes Archiv für klinische und experimentelle Ophthalmologie*.

[5] Costarides A. P., Alabata P., Bergstrom C. (2004). Elevated intraocular pressure following vitreoretinal surgery. *Ophthalmology Clinics of North America*.

[6] Kerrison J. B., Haller J. A., Elman M., Miller N. R. (1996). Visual field loss following vitreous surgery. *Archives of ophthalmology (Chicago, Ill. : 1960)*.

[7] Melberg N. S., Thomas M. A. (1995). Visual field loss after pars plana vitrectomy with air/fluid exchange. *American Journal of Ophthalmology*.

[8] Chang S. (2006). LXII Edward Jackson lecture: open angle glaucoma after vitrectomy. *American Journal of Ophthalmology*.

[9] Holló G., Zhou Q. (2016). Evaluation of retinal nerve fiber layer thickness and ganglion cell complex progression rates in healthy, ocular hypertensive, and glaucoma eyes with the Avanti RTVue-XR optical coherence tomograph based on 5-year follow-up. *Journal of Glaucoma*.

[10] Yan H., Dhurjon L., Chow D. R., Williams D., Chen J. C. (1998). Visual field defect after pars plana vitrectomy. *Ophthalmology*.

[11] Wu H., de Boer J. F., Chen T. C. (2010). Reproducibility of retinal nerve fiber layer thickness measurements using spectral domain optical coherence tomography. *Journal of Glaucoma*.

[12] Mwanza J.-C., Oakley J. D., Budenz D. L., Chang R. T., Knight O. J., Feuer W. J. (2011). Macular ganglion cell-inner plexiform layer: automated detection and thickness reproducibility with spectral domain-optical coherence tomography in glaucoma. *Investigative Ophthalmology & Visual Science*.

[13] Reddy R. K., Lalezary M., Kim S. J. (2013). Prospective retinal and optic nerve vitrectomy evaluation (PROVE) study: findings at 3 months. *Clinical Ophthalmology*.

[14] Kim K. Y., Yu S.-Y., Kim M. S., Kim E. S., Kwak H. W. (2013). Changes of parafoveal retinal nerve fiber layer thickness analyzed by spectral-domain optical coherence tomography after pars plana vitrectomy. *Retina*.

[15] Balducci N., Morara M., Veronese C., Torrazza C., Pichi F., Ciardella A. P. (2014). Retinal nerve fiber layer thickness modification after internal limiting membrane peeling. *Retina*.

[16] Gharbiya M., La Cava M., Tortorella P. (2016). Peripapillary RNFL thickness changes evaluated with spectral domain optical coherence tomography after uncomplicated macular surgery for epiretinal membrane. *Seminars in Ophthalmology*.

[17] Uemura A., Kanda S., Sakamoto Y., Kita H. (2003). Visual field defects after uneventful vitrectomy for epiretinal membrane with indocyanine green-assisted internal limiting membrane peeling. *American Journal of Ophthalmology*.

